# Complex post-traumatic stress disorder in asylum seekers and victims of trafficking: treatment considerations

**DOI:** 10.1192/bjo.2021.1007

**Published:** 2021-10-01

**Authors:** Sally Jowett, Angeliki Argyriou, Odile Scherrer, Thanos Karatzias, Cornelius Katona

**Affiliations:** Edinburgh Napier University, School of Health and Social Care, UK; Therapy, Helen Bamber Foundation, UK; School of Health and Social Care, Edinburgh Napier University, UK; and Rivers Centre for Traumatic Stress, NHS Lothian, UK; Therapy, Helen Bamber Foundation, UK; and Division of Psychiatry, University College London, UK

**Keywords:** Trauma, psychosocial interventions, CPTSD, asylum seekers, refugees

## Abstract

Asylum-seekers experience high levels of traumatic events pre-, post- and during migration. Poly-traumatisation is associated with complex post-traumatic stress disorder (CPTSD), which has not yet been extensively explored in this population. CPTSD is a prevalent and highly disabling disorder in the present population requiring culturally sensitive diagnostic and treatment approaches. In this service evaluation, we evidence the high prevalence of CPTSD in an asylum-seeking sample and its association with greater distress compared with PTSD. We outline the treatment needs of asylum seekers with CPTSD.

## Background

By the end of 2019, 79.5 million people had been forcibly displaced from their home country.^1^ That same year, there were 35 099 asylum applications in the UK from people fleeing persecution.^2^ High numbers of asylum seekers report multiple traumatic life events including war, violence, torture and human trafficking/modern slavery. Following the need to escape, the journey undertaken to arrive in their settled country is often immensely dangerous and traumatic in itself.^3^ Exposure to such traumatic events can lead to the development of a wide range of mental health difficulties, such as post-traumatic stress disorder (PTSD), which includes symptoms of re-experiencing the trauma through flashbacks or nightmares, avoidance of reminders and hyperarousal. In fact, refugees and asylum seekers have been shown to be ten times more likely to develop PTSD than the general population of their settled country.^4^

Complex PTSD (CPTSD) is a relatively new diagnosis recognised within the ICD-11 classification^5^ characterised by the core PTSD symptoms together with ‘disturbances in self-organization’ (DSO) in the form of affect dysregulation, difficulties in interpersonal relationship and negative self-concept. Poly-traumatisation has been associated more strongly with CPTSD than with PTSD.^6^ CPTSD is typically, although not exclusively, associated with multiple, prolonged experiences of interpersonal trauma.^7^ Populations who seek asylum are often victims of extreme and multiple interpersonal trauma, including state and non-state torture and prolonged childhood or domestic abuse. Many of them have histories of trafficking and exploitation, including histories of forced labour and prostitution. There is recent evidence to suggest that interpersonal trauma as well as trafficking experiences can place these individuals at increased risk of developing CPTSD.^8,9^ Furthermore, the ICD-11 proposes that trauma whereby there is an inability to escape is a specific risk factor for the development of CPTSD, and the need to escape is a key aspect of the asylum-seeker experience.^7^

Following poly-traumatisation, a number of situational stressors including insecure legal status, homelessness and destitution, racism and stigma, and difficulties integrating into their community, can propagate mental health difficulties and put these vulnerable people at risk for further traumas. Recent studies have documented the rates of CPTSD being higher than those of PTSD in settled refugee populations^10–13^ and highlighted an association between DSO symptoms and post-migration living difficulties^14^ and insecure visa status.^15^

Although there has been some research on refugee populations, the literature on ICD-11 CPTSD in asylum seekers and victims of trafficking in the UK has been limited. A recent systematic review of the literature of available treatments for adult asylum seekers and refugees concluded that there appears to be a bias of scarcity of psychology research for this at-risk population.^16^ The authors argue that research evaluating the clinical needs and available treatments for this population in ‘real-world’ clinical settings is even rarer and that the new diagnosis of CPTSD can provide an important tool in broadening the available literature.

## Aims

The primary aim of this service evaluation was to examine the rates of ICD-11 PTSD and CPTSD seen by a London-based trauma-specialist service for asylum seekers in order to better understand the clinical needs of presenting clients; this would then enable the service to assess the suitability of the existing psychological interventions in meeting these needs. We discuss these findings and outline recommendations for research in this area. We also hope that the service evaluation will add to the overall literature by helping to explore the prevalence of CPTSD in asylum seekers and victims of trafficking, highlighting key challenges in accessing and engaging with available care and identifying potential targets for treatment.

## Method

The data presented are from 101 adults from 37 different nations referred to a London-based specialist trauma service for asylum seekers. All referrals accepted to the service are adults over the age of 18 who have experienced trauma in the form of direct extreme human cruelty including torture, human trafficking and other forms of interpersonal violence. Clients taken on by the service also have ongoing legal protection needs, i.e. they do not yet have legal status to remain in the UK, as well as having primary mental health needs relating to their trauma. Table 1 includes further information on pre-migration experiences including the type of trauma and trafficking status.

**Table 1:** Clinical characteristics by International Trauma Questionnaire (ITQ) diagnostic group

Variable	All participants (*n* = 101)	By ITQ diagnosis (*n* = 77)			ANOVA/χ^2^		Column statistical differences	Effect size, (η²)/Cramers V
		None (*n* = 15)	PTSD (*n* = 11)	CPTSD (*n* = 51)	*F*	*P*		
Age, mean (s.d.)	34.56 (11.01)	29.80 (11.43)	32.91 (12.24)	35.67 (10.59)	1.73	0.18		0.044
Gender, female: *n* (%)	49 (48.5)	4 (26.7)	6 (54.5)	26 (51.0)	3.07	0.22		0.200
Victim of trafficking, *n* (%)	51 (50.5)	8 (53.3)	5 (45.5)	24 (47.1)	0.22	0.90		0.053
Type of trauma, *n* (%)								
State/non-state torture	48 (47.5)	6 (40.0)	5 (45.5)	27 (52.9)	0.86	0.65		0.105
Sexual abuse/rape	56 (55.4)	6 (40.0)	8 (72.7)	28 (54.9)	2.75	0.25		0.189
Exploitation	51 (50.5)	8 (53.3)	5 (45.5)	24 (47.1)	0.22	0.90		0.053
Domestic violence	34 (33.7)	3 (20.0)	5 (45.5)	18 (35.3)	2.00	0.37		0.161
Other extreme violence	50 (49.5)	8 (53.3)	3 (27.3)	25 (49.0)	2.04	0.36		0.163
CORE (*n* = 84), mean (s.d.)								
Total	80.79 (18.5)	63.36 (18.70)^a^	71.82 (18.26)^b^	85.98 (16.68)^c^	**8.22**	<0.001**	a < c	0.188
Well-being	11.77 (3.07)	9.50 (3.03)^a^	11.00 (3.29)^b^	12.18 (2.81)^a^	**4.78**	0.01*	a < c	0.119
Problems	35.54 (8.66)	29.93 (8.96)^a^	31.91 (7.62)^b^	37.20 (8.44)^a^	**4.95**	0.01*	a < c	0.122
Functioning	27.38 (8.36)	23.29 (8.04)	23.55 (10.07)	28.73 (7.76)	3.53	0.04*^NS^		0.090
*Risk*	5.82 (5.47)	3.93 (5.34)	5.00 (4.94)	6.14 (5.87)	0.90	0.41		0.025
ITQ (*n* = 77)), mean (s.d.)								
Re-experiencing	6.40 (1.80)	5.33 (2.53)^a^	6.45 (1.92)^b^	6.71 (1.40)^c^	**3.60**	0.03*	a < c	0.089
Avoidance	5.96 (2.07)	3.93 (2.87)^a^	5.82 (1.72)^b^	6.59 (1.40)^c^	**12.45**	<0.001**	a < c	0.252
Sense of threat	5.69 (2.29)	3.27 (2.92)^a^	5.82 (1.25)^b^	6.37 (1.74)^c^	**14.47**	<0.001**	a < b < c	0.281
Affect dysregulation	5.32 (2.09)	3.93 (1.90)^a^	5.09 (2.17)^b^	5.82 (1.94)^c^	**5.25**	0.01*	a < c	0.126
Negative self-concept	5.92 (2.56)	4.86 (2.60)^a^	2.82 (2.96)^b^	6.84 (1.76)^c^	**18.25**	<0.001**	b < a < c	0.333
Disturbed relationships	5.65 (2.42)	3.14 (3.01)^a^	4.73 (2.41)^b^	6.51 (1.63)^c^	**15.97**	<0.001**	a < b < c	0.304
PMLDC (*n* = 71)), mean (s.d.)								
Basic survival	25.32 (9.17)	20.23 (11.54)	25.0 (8.66)	26.72 (8.25)	2.64	0.08		0.075
Healthcare	7.13 (5.49)	3.38 (3.50)^a^	9.11 (5.93)^b^	7.96 (5.42)^c^	**4.61**	0.01*	a < b, c	0.124
Relationships	7.25 (3.73)	8.00 (3.29)^a^	4.33 (4.56)^b^	7.65 (3.51)^c^	**3.49**	0.04*	b < c	0.097
Integration	16.17 (4.12)	15.31 (5.51)	16.22 (4.38)	16.33 (3.68)	0.31	0.74		0.009
Housing	10.42 (6.70)	9.31 (5.74)	11.11 (9.98)	10.61 (6.39)	0.24	0.79		0.007
Total	64.39 (21.10)	56.23 (22.69)	59.3 (29.82)	68.59 (17.46)	2.27	0.11		0.064

PTSD, post-traumatic stress disorder; CPTSD, complex PTSD; CORE, Clinical Outcomes in Routine Evaluation; PMLDC, Post-Migration Living Difficulties Checklist; NS, non-significant in *post hoc* tests.

*P < 0.05; **P *<* 0.001; categorical variables analysed with χ^2^ were gender, victim of trafficking and type of trauma. a, b, c subscripts indicate which groups have statistical difference.

For the ANOVA tests, the effect size is presented as Eta Squared and for Chi-Square tests as Cramer's V.

The service routinely assesses for mental health problems, including PTSD, depression, and other common mental health problems and offers a variety of trauma-focused interventions including narrative exposure therapy (NET), trauma-focused cognitive–behavioural therapy, and eye movement desensitisation and reprocessing. Low-intensity interventions such as behavioural activation, anxiety management and grounding for dissociation and flashbacks are also offered. Although clinicians routinely work with complex presentations that may be best described by a CPTSD diagnosis, no existing treatment pathways are available for the presentation in the service.

The data were collected routinely as part of all new clients’ initial assessments between January 2019 and March 2020. These assessments are usually conducted with an interpreter. They included a clinical interview and a battery of questionnaires. Data were fully anonymised prior to analysis. The project was reviewed by the Chair of the Health Sciences Research Governance Committee, University of York who confirmed that it was a service evaluation and that it was not necessary to obtain specific consent from the clients whose data are included in the analysis.

The questionnaires used were the Clinical Outcomes in Routine Evaluation (CORE) for well-being,^17^ the International Trauma Questionnaire (ITQ) for trauma symptomatology^18^ and the Post-Migration Living Difficulties Checklist (PMLDC) for challenges faced in order to settle in the UK.^19^ There were significant levels of missing data across the questionnaires (CORE, 16.8%; ITQ, 23.8%; PMLDC, 29.7%) because of difficulties with comprehension, dissociation and risk, which required the assessment was terminated early, an important feature of working with this population.

For the statistical analysis, the groups meeting criteria for PTSD and CPTSD were compared with those without trauma symptomatology through a series of ANOVA and χ^2^-tests. Descriptive statistics were applied across the whole sample and the diagnostic subgroups, and all analyses were completed within SPSS v.27.^20^

## Results

The mean age of the participants was 34.56 years, 48.5% were female and 50.5% were identified as victims of trafficking. The most commonly reported trauma was sexual abuse/rape (55.4%). Out of those who completed the ITQ (*n* = 77), approximately two-thirds (66.23%) met criteria for ICD-11 CPTSD; this subgroup presented with significantly greater levels of distress on the CORE compared with those with no diagnosis as per ITQ criteria. A further 14.3% met criteria for PTSD. Those with PTSD and CPTSD presented with significantly higher healthcare needs than those with no PTSD, whereas those with CPTSD presented with significantly higher relationship-related needs compared with those with PTSD.

There were varying levels of missing data across each of the assessment questionnaires leading to unequal group sizes. Our main analysis used the ITQ data; the majority of participants who completed the ITQ were also able to complete the CORE (96.1%) and the PMLDC (89.6%). We therefore anticipate the impact of missing data on this part of our analysis to be minimal. We balance this limitation with the responsibility to display as much information from the data we were able to collect. To this end, the descriptive data for all participants (*n* = 101) is also included to provide a more balanced illustration of the characteristics and trauma backgrounds within the service, rather than the subset who were able to engage with the full assessment battery.

## Discussion

In this multiply traumatised, asylum-seeking sample, two-thirds (66.23%) of those with complete assessments met criteria for ICD-11 CPTSD. This finding supports previous research on the high prevalence of CPTSD in other UK clinical samples.^6^ Despite the barriers to conducting assessments with this highly complex group, over 70% of data were collected in full from all clients, which provides confidence in the representativeness of the data-set. This study has demonstrated the feasibility and necessity of auditing the mental health and social needs of an asylum-seeking population, a group that can face higher displacement, unsettled living circumstances and barriers in accessing services than settled refugee groups do and thus are less represented in literature.

In light of our findings, clinicians working with asylum seekers and victims of trafficking are encouraged to assess specifically for CPTSD as well as PTSD and to adapt existing trauma-focused treatments to address the additionally impairing DSO symptom clusters. The treatment of CPTSD requires that both PTSD and DSO symptoms should be targeted for intervention.

Multicomponent approaches have been recommended for CPTSD,^21^ with phased interventions evidenced to be especially effective for refugee and asylum-seeker populations.^22^ Our results suggest that our sample experienced high levels of CPTSD symptoms, elevated clinical distress and significant difficulties in post-migration adjustment especially in the relationships domain. With these multicomponent challenges in mind, individuals who present with CPTSD can be offered a tailor-made, formulation-driven, treatment package that includes trauma-focused therapy interventions to target the core symptoms of PTSD, as well as additional modules to address the DSO symptom clusters. For instance, interventions such as Skills Training in Affective and Interpersonal Regulation^23^ can be offered to target affective dysregulation, and there is emerging evidence that compassion-focused therapy could target difficulties in negative self-concept and shame.^24^ Moreover, it has been suggested that NET, a trauma-focused intervention that has been developed and used for asylum seekers presenting with poly-traumatisation,^16,25^ is effective in treating CPTSD, as are protocols that target the dissociative symptoms with which this client group often presents.^26^ Clinicians can formulate the most pertinent and impairing difficulties and offer appropriate interventions to tackle the most prominent symptoms^27^ also taking into account the type of trauma^28^ as well client's readiness to tackle these symptoms. Interventions offered should be culturally sensitive and adapted appropriately to lend themselves to work with people who do not speak English fluently and to work with interpreters.

Pathways for the treatment of CPTSD in this population should include considerations about length and flexibility of treatment. Treatment for CPTSD is likely to be lengthier than for PTSD, not only to address a larger number of symptoms, but also to allow for extended stabilisation work aimed at developing the rapport, trust, safety and stability that are pre-requisites for effective treatment, and addressing barriers to engagement. Stabilisation work can be offered concurrently with interventions that actively address the symptoms of CPTSD and not necessarily in a consecutive manner. Moreover, the multitude of psychosocial needs that refugees and asylum seekers present with, along with the complexity of their clinical presentation, indicates the need for a flexible, integrated and holistic treatment approach.

There is evidence to suggest that insecure visa status and ongoing threat predict exacerbation in the DSO symptoms of CPTSD.^15^ Therefore, it is paramount that psychosocial support is offered in a timely and flexible manner and in conjunction with integrated support with other areas of the person's life, including social integration, legal, housing and other systemic matters. For example, Robertson and colleagues have developed a group protocol for refugees and asylum seekers which incorporates these varied and complex needs into psychological treatment.^22^

### Limitations

This service evaluation has a number of limitations. First, there are significant levels of missing data because of the difficulties in completing self-reports at the initial assessment appointments for individuals who presented with acute levels of risk, dissociation or comprehension difficulties. Moreover, the ITQ requires identifying the most distressing trauma, which is difficult to disclose or engage in early stages of engagement with the service. Although this is an essential process to anchor symptoms to traumatic life events, this reflects the challenges in working with this vulnerable population and quantifying their complex difficulties in clinical research. Nevertheless, the existence of missing data compromises the generalisability of the findings and it may mean that some of the most complex clients are less represented in our findings. Future research could attempt to minimise this problem by scheduling breaks in assessments, having a calm and predictable environment, and minimising the number of questionnaires. However, although these are all steps that are taken in the service, the reality is that acute and high levels of distress in combination with language and communication barriers are likely to present challenges for data gathering and priority should be given to safeguarding the well-being of clients while ensuring their active, ongoing consent to complete measures.

Furthermore, our analyses are vulnerable to type I error and our findings should be interpreted cautiously. The unequal group sizes make it difficult to reliably form conclusions from these findings. For instance, it is unclear why the PTSD group were found to experience higher healthcare needs than the CPTSD group. Our results therefore require replication in larger samples in order to generate sufficient statistical power to clarify differences between types of traumas, gender and age groups. Only PTSD and CPTSD were assessed in this report; however, future research should assess for a broader range of mental health difficulties as trauma is a risk factor for many diagnoses including depression and anxiety disorders.^29^ It is also important to note that no interview measures were included; this potentially limits the clinical validity of the data as interview measures have been found to provide lower prevalence of disorder than self-report scales.^30^ Our results may therefore be an overestimate of CPTSD rates in this population. Future research could benefit the field further by including interview measures as well as collecting more detail on pre-migration and peri-migration factors. Moreover, our study includes help-seeking participants referred specifically to a trauma service that provides support to victims of cruelty with complex mental health and other post-migration needs, and therefore our estimates of PTSD and CPTSD may be elevated in comparison with the broader asylum-seeking population in the UK. Studies that recruit from non-specialised services may identify different needs.

### Implications

These limitations in part communicate the inherent challenges in facilitating research that represents the needs of vulnerable, displaced populations. In light of this, it is important to consider the potential next directions for research to support these groups. Our finding of high levels of CPTSD in asylum seekers and victims of trafficking needs replicating across other service contexts. These rates also point to the urgent need to further develop the evidence base of psychological and social interventions to meet the needs of this highly affected group. Furthermore, the rates of PTSD and CPTSD were commonly in the context of insecure legal status, homelessness, destitution, ongoing risks and difficulties accessing healthcare and supportive relationships as people work to integrate into their settled communities. The literature would benefit from a more nuanced understanding of the interplay of these factors by examining, for instance, the ways that post-migration stressors may moderate the effectiveness of psychological interventions and explore ways to mitigate their impact. Demonstrating the synergic impact that integrated psychological and social interventions can have on individual's post-traumatic reactions as well as general functioning and resilience would be important in driving the design of effective treatment pathways for this population group.

In conclusion, our service evaluation indicates high prevalence of CPTSD in asylum seekers at a specialist trauma clinic in the UK and high levels of associated distress and impairment. Considering the debilitating nature of CPTSD alongside other challenges that asylum seekers typically face, there is an urgent need for developing and testing the acceptability and efficacy of culturally sensitive, integrated, multimodal approaches to enable individuals to recover from CPTSD and establish a sense of safety in their settled country.

## Data Availability

The data that support the findings of this study are available on request from the corresponding author, S.J..
